# Osmotic stress enhances suberization of apoplastic barriers in barley seminal roots: analysis of chemical, transcriptomic and physiological responses

**DOI:** 10.1111/nph.15351

**Published:** 2018-07-28

**Authors:** Tino Kreszies, Nandhini Shellakkutti, Alina Osthoff, Peng Yu, Jutta A. Baldauf, Viktoria V. Zeisler‐Diehl, Kosala Ranathunge, Frank Hochholdinger, Lukas Schreiber

**Affiliations:** ^1^ Department of Ecophysiology Institute of Cellular and Molecular Botany University of Bonn Kirschallee 1 53115 Bonn Germany; ^2^ Crop Functional Genomics Institute of Crop Science and Resource Conservation (INRES) University of Bonn 53113 Bonn Germany; ^3^ School of Biological Sciences University of Western Australia 35 Stirling Highway Crawley 6009 Perth Australia

**Keywords:** apoplast, barley, osmotic stress, root, suberin, transcriptomics, water deficit, water transport

## Abstract

- Barley (*Hordeum vulgare*) is more drought tolerant than other cereals, thus making it an excellent model for the study of the chemical, transcriptomic and physiological effects of water deficit. Roots are the first organ to sense soil water deficit. Therefore, we studied the response of barley seminal roots to different water potentials induced by polyethylene glycol (PEG) 8000.
- We investigated changes in anatomical parameters by histochemistry and microscopy, quantitative and qualitative changes in suberin composition by analytical chemistry, transcript changes by RNA‐sequencing (RNA‐Seq), and the radial water and solute movement of roots using a root pressure probe.
- In response to osmotic stress, genes in the suberin biosynthesis pathway were upregulated that correlated with increased suberin amounts in the endodermis and an overall reduction in hydraulic conductivity (Lp_r_). In parallel, transcriptomic data indicated no or only weak effects of osmotic stress on aquaporin expression.
- These results indicate that osmotic stress enhances cell wall suberization and markedly reduces Lp_r_ of the apoplastic pathway, whereas Lp_r_ of the cell‐to‐cell pathway is not altered. Thus, the sealed apoplast markedly reduces the uncontrolled backflow of water from the root to the medium, whilst keeping constant water flow through the highly regulated cell‐to‐cell path.

## Introduction

Climate changes and extreme weather conditions, such as drought, will become more intensive in the future (Melillo *et al*., 2014). This will have a major impact on agricultural productivity. Compared with other abiotic stresses, drought accounts for the highest crop losses (Boyer, 1982). Barley (*Hordeum vulgare*) is more drought tolerant than other crop plants, and represents the fourth most abundant cereal after wheat, maize and rice (http://faostat.fao.org). Other than drought, barley is also fairly resistant to other abiotic stresses, such as salinity, alkalinity and cold, and can survive better under nonoptimal environmental conditions (Colmer *et al*., 2006; Kosová *et al*., 2014). These unique properties make barley a model crop for the study of the effect of abiotic stresses in general. Drought starts with a decrease in the soil water potential. Consequently, plant roots are the first organs which sense drought and have to cope with water deficiency (Zingaretti *et al*., 2013).

The main function of roots is water and nutrient uptake, which is highly dependent on anatomical structures, growth conditions and plant age. Water and solute uptake of plant roots is best described by the composite transport model. According to the model, there are three major pathways for water and solute transport in roots: (1) the apoplastic (cell walls), (2) the symplastic and (3) the transcellular pathway. The last two are also referred to as the cell‐to‐cell pathway. The apoplastic pathway can be blocked by Casparian bands and suberin lamellae in endodermal and exodermal cell walls. The cell‐to‐cell pathway can additionally be regulated by aquaporins (Peterson & Cholewa, 1998; Steudle & Peterson, 1998; Steudle, 2000a,b).

The formation of the biopolyester suberin has been shown to be enhanced by abiotic (Hose *et al*., 2001; Enstone *et al*., 2002; Krishnamurthy *et al*., 2009; Ranathunge *et al*., 2011a; Barberon *et al*., 2016; Kotula *et al*., 2017) and biotic (Lulai *et al*., 1998; Thomas *et al*., 2007; Ranathunge *et al*., 2008; Lanoue *et al*., 2010) stresses. The suberin lamellae contain polyaliphatic and polyaromatic domains, which are poylmerized (Kolattukudy *et al*., 1975; Bernards, 2002). The aliphatic monomers are primary alcohols, fatty acids, α–ω dicarboxylic acids (diacids) and ω‐hydroxy acids (ω‐OH acids), whereas the aromatic components are ferulic and coumaric acids (Schreiber *et al*., 1999; Graça, 2015). Casparian bands are mainly composed of lignin and partly of suberin (Schreiber, 1996; Zeier & Schreiber, 1998; Schreiber *et al*., 1999; Naseer *et al*., 2012). Lignin consists of syringyl, guaiacyl and p‐hydroxyphenol monomers which form a complex aromatic biopolymer (Fraser & Chapple, 2011; Lupoi *et al*., 2015).

Here, the effect of water deficit induced by osmotic stress through polyethylene glycol (PEG) 8000 on suberized barrier development in barley roots, and its physiological effects, are reported. Apoplastic barrier development along the root using microscopy and histochemical studies of barley roots grown under different low water potentials were investigated. Subsequently, changes in root suberization and global gene expression patterns during the different root developmental stages in response to osmotic stress were quantified. Finally, the effect of osmotic stress on water and solute transport in roots using a root pressure probe was studied. These findings indicate that an increased amount of suberin could be an effective adaptation to water deficit as a result of sealing of roots and prevention of uncontrolled passive water loss from the root to the dry soil by backflow via the nonselective apoplastic pathway. At the same time, roots maintain the uptake of water through the cell‐to‐cell pathway.

## Materials and Methods

### Plant material and growth conditions

**Figure 1:**
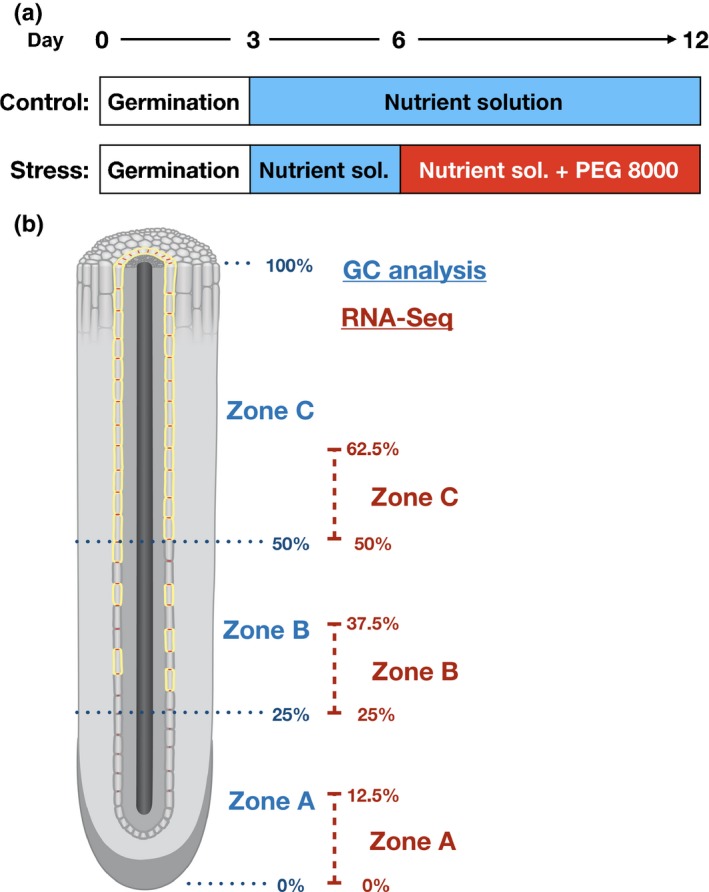
Experimental setup of long‐term osmotic stress. (a) Schematic diagram of growth conditions and low water potential application with polyethylene glycol (PEG) 8000. After 3 d of germination, seedlings were transferred to hydroponic nutrient solution. For stress treatment, the nutrient solution was exchanged with nutrient solution adjusted to a defined water potential with PEG 8000 at day 6. When the plants were 12‐d‐old, they were harvested for experiments. (b) Schematic diagram showing the different root zones which were harvested for gas chromatography (GC) analysis (blue) and RNA‐sequencing (RNA‐Seq) analysis (red). The seminal roots were divided into three zones based on the development of apoplastic barriers, such as Casparian bands and suberin lamellae. For suberin analysis by GC, three zones were selected: (1) zone A – from 0% to 25%; (2) zone B – from 25% to 50%; and (3) zone C from 50% to 100% of the total seminal root length. For RNA‐Seq analysis, the lengths of the zones were reduced to avoid an overload of material and to obtain more specific information. Here, zone A corresponds to 0–12.5%, zone B from 25% to 37.5% and zone C from 50% to 62.5% of the total seminal root length.

Seeds of barley (*Hordeum vulgare* L. spp*. vulgare* cv Scarlett) were stratified for 1 wk at 4°C. They were then germinated in the dark at 25°C covered with wet filter paper. After 3 d, seedlings were transferred into an aerated hydroponic system containing half‐strength Hoagland solution in a climatic chamber under long‐day conditions (16 h : 8 h, light : dark), an air temperature of 23°C : 20°C (day : night) and a relative humidity of 50–65%. When the plants were 6‐d‐old, stress treatment was applied for another 6 d in all experiments described; thus plants were grown for 12 d (Fig. 1a) and, at this stage, they had two leaves and five to six seminal roots.

### Water deficit application induced by osmotic stress through PEG 8000

Low water potentials were applied when the plants were 6‐d‐old (Fig. 1a). Plants were moved from half‐strength Hoagland solution (20 mOsmol kg^−1^ or −0.04 MPa of osmotic pressure) to half‐strength Hoagland solution adjusted to a defined water potential with PEG 8000 (Roth, Karlsruhe, Germany) simulating water deficit induced by osmotic stress. The water potential of the medium was reduced to −0.4, −0.8 and −1.2 MPa by adding 17.5%, 25.4% and 31.6% (w/w) PEG 8000 (Michel, 1983). The water potentials of the nutrient solutions with different levels of PEG 8000 were measured using a WP4C Water Potential Meter (Meter Group Inc., Pullman, WA, USA).

The simulation of water deficit by PEG 8000 treatment represents a widely accepted experimental approach offering various important advantages. An exactly defined and homogeneous osmotic potential acting on the roots can be adjusted. As, in nature, water stress during drought mostly occurs in a combination with heat and high light, PEG treatment allows water deficit to be examined separately (Kramer and Boyer, 1995; Verslues *et al*., 2006; Frolov *et al*., 2017). In addition, for our experiments, hydroponic culture was essential because only with this approach could root transport properties be measured using the pressure probe technique.

### Histochemical detection of Casparian bands and suberin lamellae in roots

Cross‐sections were made at 1‐cm increments along the whole seminal root using a cryostat microtome (Microm HM 500M, Microm International, Walldorf, Germany). To detect the development of Casparian bands over the root length, cross‐sections were stained with 0.1% (w/v) berberine hemisulfate for 1 h and with 0.5% (w/v) aniline blue for 30 min (Brundrett *et al*., 1988). Suberin lamellae were stained with 0.01% (w/v) lipophilic fluorol yellow 088 for 1 h (Brundrett *et al*., 1991). Cross‐sections were analyzed by epifluorescence microscopy using an ultraviolet (UV) filter set (excitation filter BP 365, dichroic mirror FT 395, barrier filter LP 397; Zeiss, Oberkochen, Germany). Photographs were taken with a Canon EOS 600D camera (Canon Inc., Tokyo, Japan at ISO 200 or 400 for 1–2 s.

### Chemical analysis of barley root suberin

The seminal roots were divided into three zones – A, B and C – based on the previous microscopic investigations (Fig. 1b). Zone A (0–25% of total root length) was the youngest part of the root, which included the root apex. In this zone, only Casparian bands were present in the endodermis, but no suberin lamellae were deposited. Zone B (25–50%) was the transition zone, in which all endodermal cells had Casparian bands, but only a limited number of cells had suberin lamellae depositions. Zone C (50–100%) was the mature part of the root close to the root base, in which all endodermal cells were characterized by the presence of Casparian bands and suberin lamellae (Fig. 1b).

For each replicate, 10 segments of seminal roots from each of the three zones were pooled together. The root segments were enzymatically digested for 3 wk with 0.5% (w/v) cellulase and 0.5% (w/v) pectinase at room temperature under continuous shaking (Zeier & Schreiber, 1997). The enzyme solution was replaced four times within the 3 wk and roots were vacuum infiltrated with the solution. Subsequently, isolated cell walls were washed in borate buffer and then transferred to 1 : 1 (v/v) chloroform : methanol for soluble lipid extraction at room temperature under continuous shaking for 2 wk. The chloroform : methanol solution was replaced four times. Finally, samples were dried on polytetrafluoroethylene (PTFE) in a desiccator containing activated silica gel. The dried samples were subjected to transesterification with BF_3_–methanol to release suberin monomers (Kolattukudy & Agrawal, 1974). Gas chromatographic analysis and mass spectrometric identification were performed as described earlier (Zeier & Schreiber, 1997, 1998). Suberin amounts were referred to the endodermal surface area. The endodermal area was calculated for each root zone: *A* = 2π · *r* · *L* (*r*, endodermis radius; *L*, length of the individual root zone). Three biological replicates were used for each experiment.

### RNA isolation

For RNA isolation, five seminal roots from five 12‐d‐old barley plants grown under control or −0.8 MPa osmotic stress conditions were pooled. Samples of each of the three root zones were taken for specific transcriptome analysis. In contrast with samples taken for chemical analysis, only half of each zone was collected (Fig. 1b). The samples were collected in 2‐ml reaction tubes with sterile steel beads inside. The samples were frozen in liquid nitrogen and ground with a mixer mill (Retsch MM400; Retsch GmbH, Haan, Germany) at a frequency of 30 rounds s^−1^ for 1 min. RNA was isolated with the RNeasyPlus Universal Mini Kit (Qiagen, Venlo, the Netherlands). RNA quality was analyzed via a NanoDrop (Thermo Fischer Scientific, Wilmington, Delaware, USA) and Agilent RNA 6000 Nano Chip (Agilent Technologies, Santa Clara, CA, USA) Bioanalyzer. For all samples, a RNA integrity number ≥ 9.1 was detected. Four biological replicates were used for each experiment.

### Processing of raw reads and analysis of differentially expressed genes

Raw sequencing data of 100‐bp paired‐end reads, obtained with an IlluminaHiSeq 4000 sequencer (BGI Tech Solutions, Hong Kong, China), were processed with CLC Genomics Workbench v.10.0.1 (https://www.qiagenbioinformatics.com/) for further analyses. After quality trimming for low‐quality scores and ambiguous nucleotides, only reads with a length of > 40 bp were retained for mapping. These reads were mapped to the barley reference genome, EnsemblPlants: Hv_IBSC_PGSB_v2, v.2.36 (Mascher *et al*., 2017, ftp://ftp.ensemblgenomes.org/pub/plants/release-36/fasta/hordeum_vulgare/dna/) allowing large gaps of up to 50 kb to span introns. Only reads that matched uniquely with ≥ 80% of their length and an identity of ≥ 90% to the reference genome were considered as mapped. Stacked reads, that is, read pairs that have identical start and end coordinates and orientation, were merged into one. Subsequently, the remaining reads were mapped to the high‐confidence annotation of the genome sequence (Mascher *et al*., 2017, ftp://ftp.ensemblgenomes.org/pub/plants/release-36/gff3/hordeum_vulgare/; v2.36). Sequences had to match with ≥ 90% of their length and ≥ 90% similarity to the set of high confidence gene models. Reads with more than one hit were excluded from subsequent read counting. Before differential expression analysis, read counts were normalized by sequencing depth and log_2_‐transformed to meet the assumptions of a linear model. Furthermore, the mean–variance relationships were estimated and used to assign precision weights to each observation to adjust for heteroscedasticity (Law *et al*., 2014). To test the quality of the data, samples were clustered in a multidimensional scaling plot (MDS plot) using the plotMDS function implemented in the Bioconductor package limma (Smyth, 2005) in R (R v.3.4.0, limma_3.32.2). Distances between sample pairs were displayed as the leading log_2_ fold changes (log_2_FC), which are defined as the estimated root‐mean‐square deviation for the top 500 genes with the largest SD among all samples. This analysis provided a visual representation of sample relationships by spatial arrangement. To assess differences in gene expression between osmotic stress treatment and control in each root tissue, a linear model including a fixed effect for treatment and tissue and an interaction effect was applied. An empirical Bayes approach was used to estimate the variability over all genes in the fitted model and to shrink the variances towards a common value (Smyth, 2004). The contrast.fit function of the R package limma was used to compute pairwise comparisons between osmotic stress treatment and control for each tissue. To correct calculated *P* values of the performed pairwise *t*‐tests for multiplicity, the false discovery rate (FDR) was adjusted to ≤ 5% according to Benjamini & Hochberg (1995). Transcripts per million (TPM) for each gene (Supporting Information Table S1) were calculated according to Wagner *et al*. (2012). The raw sequencing data have been deposited at the National Center for Biotechnology Information (NCBI) sequence read archive (SRA accession: SRP136092).

### Functional annotation and gene ontology (GO) analysis

Annotations were retrieved from EnsemblPlants (Kersey *et al*., 2016; http://plants.ensembl.org/index.html) and the IPK Barley Blast server (Deng *et al*., 2007; http://webblast.ipk-gatersleben.de/barley_ibsc/downloads/). AgriGO v.2.0 (Tian *et al*., 2017) was used for singular enrichment analysis (SEA) by comparing the list of differentially expressed genes with the customized annotated reference from the IPK Barley Blast server. The cross‐comparison of the SEA (Seacompare) tool was used to combine the SEA results.

Putative barley orthologs of suberin, lignin, fatty acid elongation and aquaporin genes are based on known mutants described in *Arabidopsis* and rice (Fraser & Chapple, 2011; Ranathunge *et al*., 2011b; Li‐Beisson *et al*., 2013; Vishwanath *et al*., 2015; Kreszies *et al*., 2018). The barley genes were retrieved via the IPK Barley Blast server (Deng *et al*., 2007) and the orthologous search from EnsemblPlants (Kersey *et al*., 2016).

### Root pressure probe experiments

Root pressure probe experiments were conducted with the end segments/apical part of the seminal roots lacking lateral roots (zone A and zone B together) as described earlier (Steudle *et al*., 1987; Ranathunge *et al*., 2017). The measurements were only performed for plants grown in control and −0.8 MPa treatment conditions. Plants grown in −0.8 MPa PEG 8000 solution were transferred back to half‐strength Hoagland nutrient solution at least 1 h before root pressure probe measurements. Between 2 and 4 h after fixing to the pressure probe, roots reached the steady‐state root pressure. In the hydrostatic experiments, water flow was induced by moving the micrometer screw forward and backward, and thus inducing radial water flow out of or into the root. The subsequent pressure changes were used to calculate the hydraulic conductivity (Lp_r_) of the roots from the half‐times of water exchange ($t_{1/2}^{w}$):

$$k_{\mathrm{wr}}=\frac{\log_{e}\left(2\right)}{t_{1/2}^{w}}={\text{Lp}}_{r}\times A_{r}\times \beta$$

β (MPa m^−3^) is the elastic coefficient of the measuring system. It was measured by inducing step changes in the volume, which result in changes in the root pressure. *A*_r_ is the surface area of the root segment mounted on the pressure probe. The hydraulically isolated nonconductive part of the root was *c*. 15 mm from the root apex.

For the osmotic experiments, the nutrient solution was rapidly exchanged with nutrient solution containing 30 mM NaCl (60 mOsmol kg^−1^). To minimize the effect of unstirred layers, the medium was stirred with aeration during all experiments. The changes in root pressure in response to the osmotic change in the medium were biphasic. A rapid water phase was followed by a slower solute phase. The water phase was used to calculate the osmotic hydraulic conductivity of the root. The solute phase § was used to calculate solute permeabilities (*P*_sr_) of NaCl:

$$k_{\mathrm{sr}}=\frac{\log_{e}\left(2\right)}{t_{1/2}^{s}}=\frac{A_{r}\times P_{\mathrm{sr}}}{V_{x}}$$

*k*_sr_ is the rate constant of permeation of solutes. Here $t_{1/2}^{s}$ is the half‐time of solute exchange and *V*_x_ is the volume of functional xylem in the root. It was 1.5% measured in the cross‐sections of seminal roots. The total root volume was calculated with the conductive root length and the root diameter. Reflection coefficients (σ_sr_) of NaCl were calculated with:

$$\sigma_{\mathrm{sr}}=\frac{\Delta P_{r}}{\Delta \pi_{s}^{\circ }}\exp\left(k_{\mathrm{sr}}\times t_{\min}\right)$$

Δ*P*_r_ is the maximum change in root pressure and *t*_min_ is the time which is required to reach the minimum root pressure. Δπ^0^_s_ is the change in the osmotic pressure of the medium, which is calculated as Δπ^0^_s_ = *R*·*T*·*C*_s_, with *R* = universal gas constant, *T* = absolute temperature and *C*_s_ = osmolarity of the solute (60 mOsmol kg^−1^).

At the end of each measurement, roots were cut close to the seal to check the proper fixation of the root: if the root pressure did not drop rapidly down to zero and if there was no drastic decrease in $t_{1/2}^{w}$ to approximately one order of magnitude faster than during hydrostatic pressure relaxations, the roots were discarded. This usually happens as a result of overtightening of the roots at the fixing point of the pressure probe that blocks the xylem vessels.

### Statistical analysis of chemical and physiological data

Data analysis and statistical tests were performed with origin Pro 9. Normal distribution of the data was tested with the Shapiro–Wilk test. As all data were normally distributed, we tested for statistical significance of differences between means of plants grown under different water potentials at a significance level of 0.05: two‐sample *t*‐test for root pressure probe experiments and analysis of variance (Fisher's least significant difference, LSD) for chemical analyses.

## Results

### Root morphology and anatomy

**Figure 2:**
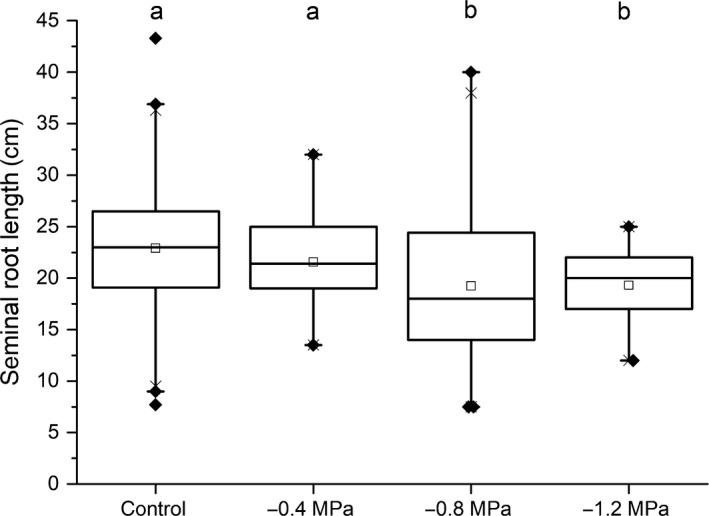
Root lengths of 12‐d‐old barley plants grown under control conditions or at a water potential of −0.4, −0.8 or −1.2 MPa. The boxes range from the 25^th^ to 75^th^ percentiles. The square in the box represents the mean value. The whiskers range to the outliers. Each box represents > 150 individual seminal roots. Different letters indicate significant differences between means at a significance level of 0.05 in one‐way ANOVA (Fisher's least significant difference, LSD).

The average length of 12‐d‐old barley seminal roots decreased with increasing osmotic stress treatments (−0.4, −0.8 and −1.2 MPa) (Fig. 2). The reduction in root length at −0.4 MPa (21.5 ± 4.0 cm) was not statistically significantly different from control conditions (22.9 ± 5.5 cm), whereas the root length was significantly reduced at −0.8 MPa (19.2 ± 6.9 cm) and −1.2 MPa (19.3 ± 3.6 cm). The seminal root length was not significantly different for the two lowest water potential treatments of −0.8 and −1.2 MPa (Fig. 2).

**Figure 3:**
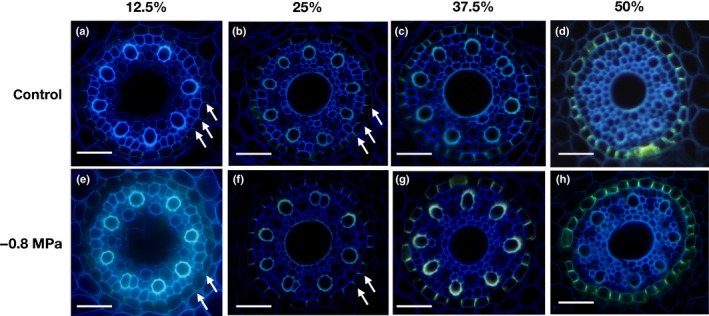
Development of Casparian bands in the endodermis of barley seminal roots in different root zones (Fig. 1b). Casparian bands of roots grown under (a–d) control conditions and (e–h) in the presence of −0.8 MPa were stained with berberine aniline sulfate. The presence of Casparian bands is indicated by yellow fluorescence. At a distance of 12.5%, thin Casparian bands are visible (arrows), which increase in length and fluorescence intensity going from 25%, via 37.5%, to 50% relative root length. Bars, 50 μm.

Endodermal Casparian bands were visible even near the root apex as small dot‐like structures (Fig. 3a,e). Starting at 12.5% of the root length, they developed to continuous bands in the radial cell wall (Fig. 3). There were no obvious differences between the control (Fig. 3a–d) and water‐stressed plants (−0.8 MPa) in the development of Casparian bands (Fig. 3e–h). Casparian bands were not detected in the rhizodermis of control and water‐deficit plants, even in the older root zones, where Casparian bands were completely developed in the endodermis. Thus, barley seminal roots fail to develop an exodermis, even under osmotic stress conditions.

**Figure 4:**
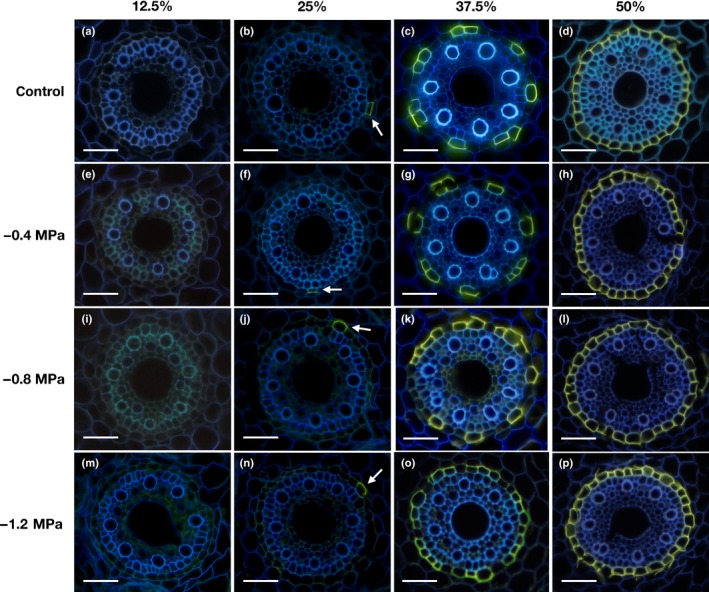
Development of suberin lamellae in the endodermis of barley seminal roots. Suberin lamellae in different zones (Fig. 1b) of roots grown under different water potentials were stained with fluorol yellow 088. The presence of suberin lamellae is indicated by a bright yellow fluorescence. At a distance of 12.5%, no suberin lamellae are visible (a, e, i, m). At 25% of relative root length, the first single, only partially suberized, cells (arrows) are visible (b, f, j, n). At 37.5% of relative root length, a patchy suberization is visible, which is stronger in roots grown in the presence of (k, o) −0.8 MPa and −1.2 MPa compared with (c) control and (g) −0.4 MPa. At a distance of 50%, the endodermis is complete suberized in all growth conditions (d, h, l, p). Bars, 50 μm.

The suberin lamellae in the endodermis started to deposit further back from the root tip than the Casparian bands and were not detectable at 12.5% of the total root length (Fig. 4a,e,i,m). In control and all osmotic stress treatments, the first appearance of single suberized cells was observed at 25% of the root length (Fig. 4b,f,j,n). At 37.5% of the total root length, there was patchy development of suberization detected in the endodermis of both control and osmotic stress treatments (Fig. 4c,g,k,o). At higher osmotic stress levels of −0.8 and −1.2 MPa, the number of suberized cells in the endodermis was higher than in the control (Fig. 4k,o). At 50% of the root length, the endodermal cells were fully suberized (complete ring of suberized cells) in control and all osmotic stress treatments (Fig. 4d,h,l,p).

### Chemical analysis of suberin of barley seminal roots in response to different osmotic stress levels

**Figure 5:**
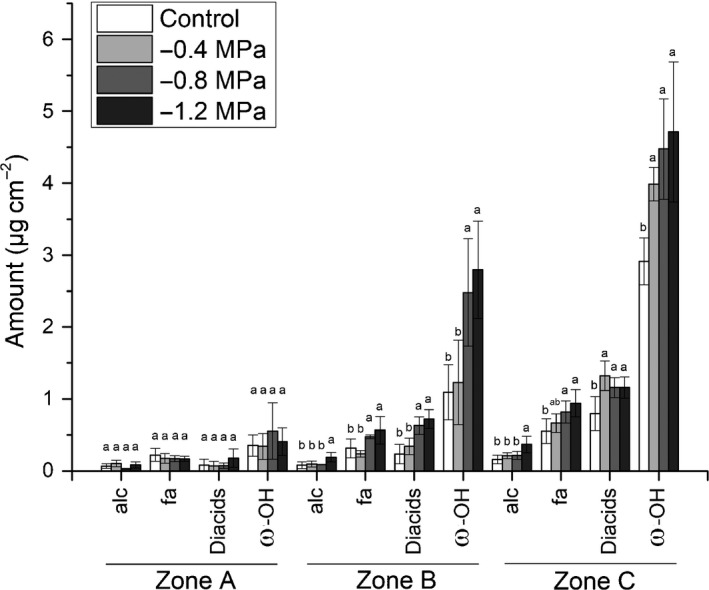
Amounts of substance classes of aliphatic suberin detected in barley seminal roots grown under control conditions or at water potentials of −0.4, −0.8 or −1.2 MPa. The roots were divided into three root zones from the apical root tip zone A over zone B to the basal part zone C. The substance classes are primary alcohols (alc), fatty acids (fa), α–ω dicarboxylic acids (diacids) and ω‐hydroxy acids (ω‐OH). The bars represent mean values with ± SD of three biological replicates. Different letters indicate significant differences between means at a significance level of 0.05 in one‐way ANOVA (Fisher's least significant difference, LSD).

**Figure 6:**
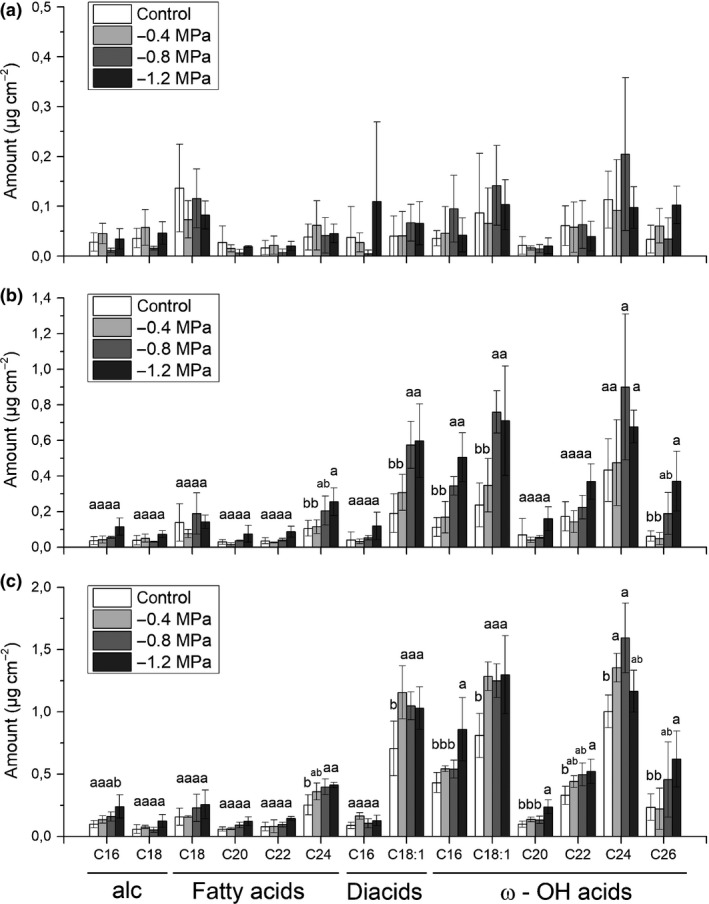
Amounts of monomers of aliphatic suberin detected in barley seminal roots grown under control conditions or at a water potential of −0.4, −0.8 or −1.2 MPa. The roots were divided into three root zones from the apical root tip (a) zone A over (b) zone B to the basal part (c) zone C. The bars represent mean values with ± SD of three biological replicates. Different letters indicate significant differences between means at a significance level of 0.05 in one‐way ANOVA (Fisher's least significant difference, LSD). In (a) zone A, no significant difference were detected. alc, primary alcohols; ω‐OH, ω‐hydroxy acids.

For chemical suberin analysis, barley seminal roots were divided into the three zones A, B and C (Fig. 1b) based on endodermal suberization (Fig. 4). Aliphatic suberin in barley seminal roots was composed of the four monomer classes: alcohols (alc), fatty acids (fa), α–ω‐dicarboxylic acids (diacids) and ω‐hydroxy acids (ω‐OH acids) (Fig. 5). The most abundant aliphatic suberin monomers were the C_18:1_ diacid and ω‐OH acids (C_18:1_ and C_24_ ω‐OH acids) (Figs 5, 6). The chain length of the different suberin monomers varied from C_16_ to C_26_ (Fig. 6). Aromatic suberin components were composed of coumaric and ferulic acids (Fig. S1). There were no significant differences in substance classes (Fig. 5) or single monomer composition (Fig. 6) between control and osmotic stress conditions.

**Figure 7:**
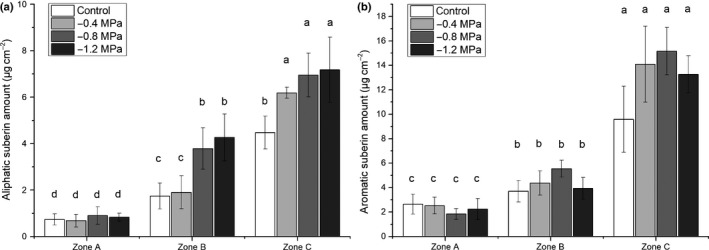
Total amounts of (a) aliphatic and (b) aromatic suberin in barley seminal roots grown under control conditions or at a water potential of −0.4, −0.8 or −1.2 MPa. The roots were divided into three root zones from the apical root tip zone A over zone B to the basal part zone C. The bars represent mean values with ± SD of three biological replicates. Different letters indicate significant differences between means at a significance level of 0.05 in one‐way ANOVA (Fisher's least significant difference, LSD).

However, the absolute (Figs 5, 6) and relative (Fig. S2) amounts of substance classes changed over the length of the root from zone A to zone C in all treatments (control and osmotic stress conditions). This change was pronounced, in particular, for the total amounts of aliphatic (Fig. 7a) and aromatic (Fig. 7b) suberin. Barley seminal roots showed a significant increase in total aliphatic and aromatic suberin (Fig. 7a,b) from zones A to C (Fig. 5), which correlated well with the suberin histochemical observations (Fig. 3). Comparing the severity of osmotic stress treatments with the degree of aliphatic suberization, there was no significant difference between treatments in zone A (Fig. 7a). In zone B, mild osmotic stress (−0.4 MPa) did not significantly enhance suberization in comparison with the control. However, stronger osmotic stress treatments of −0.8 MPa and −1.2 MPa increased the aliphatic suberin amounts by two‐fold compared with the control and −0.4 MPa (Fig. 7a). In zone C, all water stress treatments significantly increased the aliphatic suberin amounts compared with the control, but there was no significant difference between the treatments (Fig. 7a). In contrast with the total aliphatic suberin (Fig. 7a), the total aromatic suberin content significantly increased from zones A to C, but there were no significant differences between control and osmotic stress treatments (Fig. 7b). In the control, the total aromatic suberin amount was two‐fold higher than aliphatic suberin, but this ratio decreased under water stress, because of the increase in aliphatic suberin (Fig. 7).

The increase in aliphatic suberin between the three zones was mainly a result of increases in the amounts of diacids and ω‐OH acids (Figs 5, 6). For example, the amount of alcohols and fatty acids in zone C was twice the amount of zone A, but this was a ten‐fold increase for diacids and ω‐OH acids (Fig. 5). In osmotic stress treatments, this increment was even more pronounced, with a 12‐fold increase in diacids and ω‐OH acids in zone C compared with zone A (Fig. 6). The relative amounts of fatty acids and alcohols decreased from 33% and 9% in zone A to 12% and 4% in zone C, respectively, whereas the diacids and ω‐OH acids increased from 9% and 49% in zone A to 18% and 66% in zone C, respectively (Fig. S2).

### Transcriptome analysis of barley seminal roots using RNA‐sequencing (RNA‐Seq)

To identify global gene expression changes in barley seminal roots with respect to suberin development, total RNA was extracted from the three root zones (A, B and C) from control and −0.8 MPa conditions (Fig. 1b) and subjected to RNA‐Seq. We chose a water potential of −0.8 MPa for the stress treatment, because the responses of roots for growth and suberization were more pronounced compared with −0.4 MPa, but not different from the treatment with −1.2 MPa (Figs 2, 4, 7).

**Figure 8:**
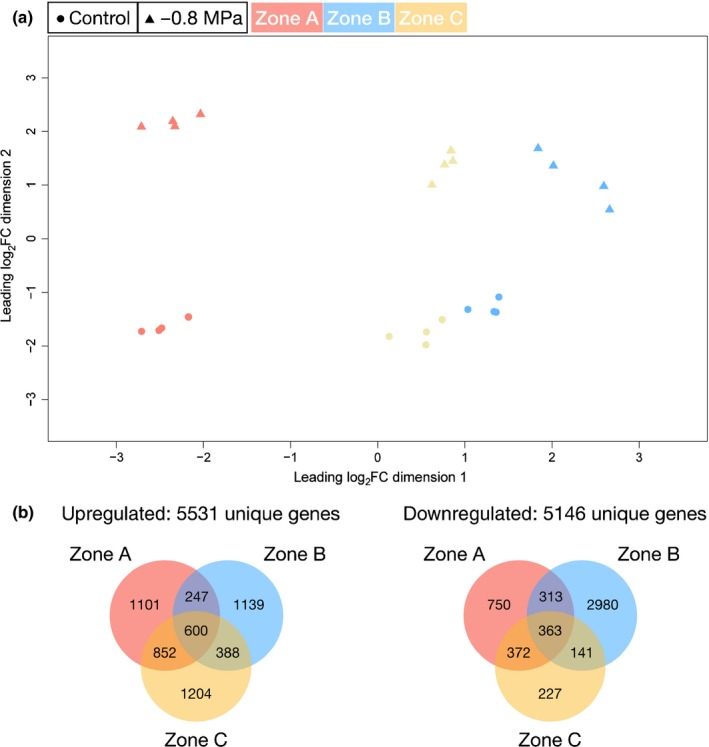
(a) Multidimensional scaling plot of barley seminal root zones grown under control conditions or at a water potential of −0.8 MPa. The roots were divided into three root zones from the apical root tip zone A over zone B to the basal part zone C. Dots, control; triangles, −0.8 MPa; red, zone A; blue, zone B; yellow, zone C. (b) Numbers of differentially expressed genes in barley root zones in response to osmotic stress. Overlap of the 5531 upregulated genes. Overlap of the 5146 downregulated genes.

RNA‐Seq yielded, on average, 35 million reads for each of the four biological replicates per zone by treatment combination. In an MDS plot, the replicate samples of the three root zones and the control vs stress conditions clustered separately, and were thus clearly distinguishable (Fig. 8a). The analysis of differentially expressed genes with FDR ≤ 5% showed that, in total, 5531 unique genes were upregulated and 5146 unique genes were downregulated. However, the response to osmotic stress was also root zone specific with 1101, 1139 and 1204 unique upregulated genes and 750, 2980 and 227 unique downregulated genes in zones A, B and C, respectively (Fig. 8b; Table S2). Functional categorization was performed using preliminary annotated barley GO terms from the IPK Barley Blast server (Deng *et al*., 2007), and the identification of significantly enriched GO terms by singular enrichment analysis with AgriGO v.2 (Tian *et al*., 2017). The analysis showed 95 unique enriched GO terms when comparing the differentially expressed genes between the three root zones under control and stress conditions (Table S3). Significantly enriched biological processes in response to osmotic stress shared by the three root zones were (1) organic acid metabolic process, (2) carboxylic acid metabolic process and (3) oxoacid metabolic process (Table S3).

**Figure 9:**
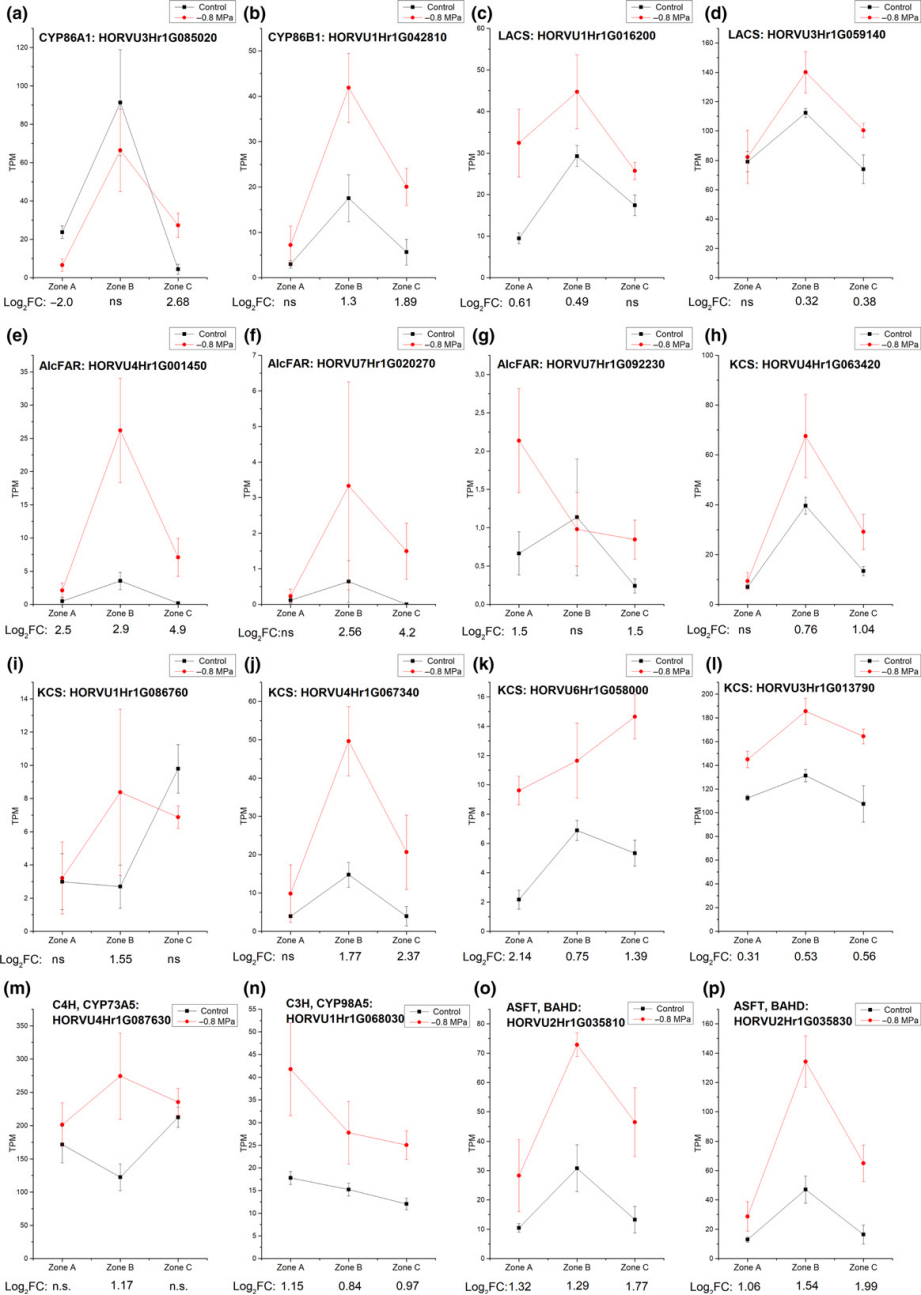
Expression patterns of most highly upregulated suberin biosynthesis genes in barley roots obtained by RNA‐sequencing (RNA‐Seq). The roots were divided into three root zones from the apical root tip zone A over zone B to the basal part zone C. Transcripts per million (TPM) for the root zones A, B and C of selected genes and their log_2_FC in response to osmotic stress are given. The log_2_FC values are given when control and PEG 8000‐treated roots display significantly different expression levels at a significance level of 0.05 in pairwise *t*‐tests. ns, not significant. (a, b) Cytochromes P450 converting fatty acids into ω‐hydroxy acids and α–ω dicarboxylic acids. (c, d) Long‐chain acyl‐CoA synthetases (LACS). (e–g) Alcohol‐forming fatty acyl‐CoA reductase (AlcFAR). (h–l) Ketoacyl‐CoA synthase (KCS) from the fatty acid elongation complex. (m, n) Cytochromes P450 synthesizing coumaric and ferulic acids. (o, p) Aliphatic suberin feruloyl transferase linking aliphatic and aromatic suberin monomers to suberin building units (ASFT/BAHD).

A significant upregulation of barley suberin genes in control as well as in −0.8 MPa treatments was detected in all three root zones (Fig. 9). In most cases, the highest expression was in zone B (Fig. 9). In total, more suberin genes were upregulated in zones B and C with higher log_2_FC values compared with zone A (Fig. 9). On average, the expression of aquaporin genes was 50 times higher than barley suberin‐associated genes in barley roots. In addition, in contrast with suberin genes, the expression of the majority of barley aquaporin genes was not significantly different in response to osmotic stress, in which few genes were up‐ or downregulated. Only HORVU1Hr1G047100, a putative NIP5;1 ortholog (portable aquaporin for boric acid and water), was highly upregulated in all three root zones (Table S4). Genes from the phenylpropanoid pathway, which are involved in the biosynthesis of lignin, which is part of the composition of Casparian bands and is heavily deposited in the central cylinder of roots, were also found to be upregulated (Table S4).

### Hydraulic conductivity, solute permeability and reflection coefficient of barley seminal roots in response to osmotic stress

Similar to the RNA‐Seq analysis, we chose a water potential of −0.8 MPa to compare the hydraulic conductivity (Lp_r_) and solute permeability of barley seminal roots between control and osmotic stress conditions (Table 1). The hydrostatic Lp_r_ was significantly reduced by 2.5‐fold (from 8.11 × 10^−8^ to 3.19 × 10^−8^ m s^−1^ MPa^−1^) in response to osmotic stress. By contrast, the osmotic Lp_r_ did not change in response to osmotic stress (Table 1). Thus, the ratios of hydrostatic : osmotic Lp_r_ declined in the osmotic stress treatment and showed that there is a shift of water flow from the apoplastic pathway to the cell‐to‐cell pathway during osmotic stress treatment (−0.8 MPa).

**Table 1:** Hydrostatic and osmotic hydraulic conductivity (Lp_r_), solute permeability (P_sr_) and reflection coefficient (σ_sr_) for NaCl of individual barley seminal roots grown under control or osmotic stress conditions (water potential of −0.8 MPa)

Parameter	Control	−0.8 MPa (osmotic stress)
Hydrostatic Lp_r_ (10^−8^ m s^−1^ MPa^−1^)	8.11 ± 2.37a	3.19 ± 1.45b
Osmotic Lp_r_ (10^−8^ m s^−1^ MPa^−1^)	3.15 ± 3.0a	3.59 ± 1.91a
Hydrostatic/osmotic	4.27 ± 2.58a	1.11 ± 0.36b
Solute permeability P_sr_ (10^−9^ m s^−1^)	2.24 ± 1.54a	0.61 ± 0.61a
Reflection coefficient (σ_sr_)	0.38 ± 0.06a	0.38 ± 0.17a

Values are given as means ± SD of eight independent replicates (*n *=* *8). Different letters indicate significant differences at a significance level of 0.05, analyzed using a two‐sample *t‐*test.

The solute permeability (P_sr_) of roots for NaCl was also reduced by the osmotic stress treatment compared with the control, but was not statistically significant because of the high variability among the water‐stressed roots (Table 1). There was no change in the reflection coefficient (σ_sr_) for NaCl in response to osmotic stress treatment compared with the control (Table 1).

## Discussion

Plant roots are the first organs to sense water deficit in dehydrating soil and thus play a crucial role in plant drought responses. In this approach, multifaceted techniques were used to test the hypothesis that an increased suberization of barley roots could represent an efficient response to water deficit by limiting uncontrolled, passive water loss from roots to the dry soil. By adding different concentrations of PEG 8000 to the nutrient solutions of hydroponically growing barley plants, specific water potentials from mild (−0.4 MPa) to more severe (−0.8 and −1.2 MPa) water deficit were adjusted.

One of the most important parameters in seedling root system architecture in response to osmotic stress is the seminal root length, because barley seminal roots contribute to overall root water uptake during early development (Knipfer & Fricke, 2010). At more negative water potentials of −0.8 and −1.2 MPa, barley roots developed 10% significantly shorter seminal roots compared with control and mild osmotic stress treatment (−0.4 MPa) (Fig. 2). This phenotypic alteration in seminal roots is probably a result of osmotically driven reduced cell elongation and organ development in declining water potentials (Yamaguchi & Sharp, 2010), resulting in reduced root length.

Detailed knowledge of the anatomy of the developmental stages along the root was important for our further analyses, including chemical, transcriptomic and water transport measurements and their interpretations (Steudle & Peterson, 1998; Steudle, 2000b; Kreszies *et al*., 2018). The suberin lamellae were only visible in the endodermis and we detected no exodermis, not even under the most severe osmotic stress conditions (−1.2 MPa) applied. This is very different from other crop plants, such as rice and maize, which develop a strong exodermis in response to stress (Schreiber *et al*., 2005; Ranathunge *et al*., 2011a, 2016). Our results on barley seminal root anatomy are consistent with previous studies (Knipfer & Fricke, 2011; Ranathunge *et al*., 2017).

In the youngest root zone (0% and 12.5% from the root tip), suberized cells were never detected (Fig. 4a,e,i,m) and only Casparian bands were visible in some instances. The first single suberized cells appeared at the border of zone A to zone B at 25% (Fig. 4b,f,j,n). At the beginning of 50% of the root length, > 90% of the endodermal cells were suberized (Fig. 4d,h,l,p). The histochemical observations show that barley roots undergo strong suberization in response to osmotic stress (Fig. 4), which was observed previously in plant roots as a general response towards abiotic stresses (Hose *et al*., 2001; Enstone *et al*., 2002; Krishnamurthy *et al*., 2009, 2011; Ranathunge *et al*., 2011b; Shiono *et al*., 2014a; Barberon *et al*., 2016; Tylová *et al*., 2017). Nevertheless, histochemical studies on suberization only provide a qualitative picture of root developmental status, whereas direct analytical methods, such as gas chromatography and mass spectrometry, can be used for the quantification of suberin amounts (Schreiber *et al*., 2005).

Suberin monomers obtained after transesterification belonged to fatty acids, alcohols, ω‐OH acids and diacids (Fig. 5). Aromatic monomers consisted of coumaric and ferulic acid (Fig. S1). This is in accordance with typical suberin compositions described in the literature (Kolattukudy & Agrawal, 1974; Bernards, 2002; Ranathunge *et al*., 2011b; Graça, 2015). In contrast with aliphatic suberin monomers, the results of much greater amounts of aromatic monomers (coumaric and ferulic acid) should be interpreted cautiously, because they can also be bound to all other cell walls in Graminaceae species (Carpita, 1996). The suberin monomer composition under control conditions in this study (Figs 5, 6) is comparable with that of a previously described suberin composition in the barley cultivar Golf (Ranathunge *et al*., 2017), suggesting that suberin monomer composition is well conserved in barley roots, even under osmotic stress conditions.

Our chemical analysis confirmed the increase in root suberization along the root and in response to osmotic stress (Fig. 7), also observed by microscopy (Fig. 4). A very low suberization was observed in zone A (0–25%). This is consistent with the observation of the first single suberized cells appearing at the border of zone A to zone B at 25%. However, in the distal half of zone A (0–12.5%), only Casparian bands were detectable in some instances (Fig. 3) and suberin lamellae have never been found with fluorol yellow 088 staining in this root zone (Fig. 4). Interestingly, our transcriptomic data clearly showed that suberin biosynthesis genes were expressed in this youngest root zone (Fig. 9). Either fluorol yellow 088 staining may not be sufficiently specific to detect very thin suberin lamellae in this zone or the measured suberin monomers are derived from Casparian bands. A third possibility, which cannot be excluded at the moment, is that histochemically undetectable suberin lamellae are synthesized and deposited somewhere else in the cell walls in this youngest root zone (0–12.5%), which might explain why suberin biosynthesis genes are upregulated in this zone.

Nevertheless, this observation is of major interest as there is an ongoing debate as to whether the chemical composition of Casparian bands is exclusively pure lignin or a mixture of lignin as the major component and suberin occurring in minor amounts. In isolated Casparian bands of *Clivia miniata*,* Monstera deliciosa*, soybean, pea and maize, mainly lignin, but also suberin, was detected by GC‐MS analyses (Karahara & Shibaoka, 1992; Schreiber *et al*., 1994, 1999; Schreiber, 1996; Zeier & Schreiber, 1997, 1998; Zeier *et al*., 1999; Thomas *et al*., 2007). Indeed, just recently, direct Raman scattering microscopic investigations of Casparian bands in maize roots reported that they are composed of both polymers, lignin and suberin (Man *et al*., 2018). However, it was concluded from promoter β‐glucuronidase (GUS) assays of suberin genes with specific endodermal expression in *Arabidopsis* roots that Casparian bands are exclusively composed of lignin, but not suberin (Naseer *et al*., 2012).

A final conclusion regarding the presence or absence of suberin as an additional polymer in Casparian bands cannot be drawn at the moment for barley roots, as different results have been obtained from different species and different experimental approaches. Caution should be exercised when transferring results obtained from *Arabidopsis* to other plant species, including crop plants. Such simple and direct one‐to‐one correlations may not always be valid (Kreszies *et al*., 2018). However, future experimental approaches with higher resolution, allowing, for example, the direct analysis of the chemical composition of Casparian bands of *Arabidopsis* roots, might help to answer this question. Alternatively, the best option would be an endodermis‐specific transcriptomic analysis by RNA‐Seq, in combination with chemical analyses of isolated and purified endodermal cell walls, which would provide a higher sensitivity and accuracy than qualitative histochemical staining techniques.

The results of our RNA‐Seq analysis in barley roots displayed root zone‐specific differential gene expression in response to osmotic stress. This is in agreement with the recently published data for maize and rice roots (Shiono *et al*., 2014b; Opitz *et al*., 2016). It was obvious that transition zone B (25–37.5%) showed the highest expression of suberin biosynthesis genes in barley roots for both control and osmotic stress conditions (Fig. 9). This confirmed the microscopic observations (Fig. 4) and chemical analyses (Fig. 7), indicating that, in zone B, there was a rapid and pronounced increase in endodermal suberization. In response to the adaptation to water stress (–0.8 MPa), suberin genes were often significantly up‐regulated in zone B compared with the control (Fig. 9), leading to faster and greater root suberization. This can be interpreted as a strategy of the root to efficiently block the apoplastic pathway, preventing uncontrolled water losses from the root to the surrounding medium/soil.

During the developmental transition of the root from zone A to B, there was a pronounced shift in suberin monomer composition from monofunctional fatty acids to ω‐OH and diacids (Fig. S2). This can also be explained by the higher expression of suberin biosynthesis genes, such as HORVU3Hr1G085020 and HORVU1Hr1G042910, which are directly located after fatty acid synthesis in the suberin biosynthesis pathway (Figs 9, S3). In zone C, in which the highest amount of suberin (Fig. 7) and a completely suberized endodermis were detected (Fig. 4), the expression of suberin biosynthesis genes was lower than in zone B, but not completely turned off (Fig. 9). Our data show that there is a maximum amount of about 7 μg cm^−2^ of aliphatic suberin in barley seminal roots in response to osmotic stress (Fig. 7). As roots failed to develop an induced exodermis in barley under osmotic stress, the endodermal suberin is attributed to the total root suberin. This amount is more than double the amount of *Arabidopsis* suberin (1.5–3 μg cm^−2^) (Ranathunge & Schreiber, 2011), but still lower than the endodermal suberin measured in rice under different abiotic stress conditions (8–12.5 μg cm^−2^) (Schreiber *et al*., 2005; Ranathunge *et al*., 2011a, 2016).

In drying soils, it is a major advantage for plants to increase suberization in the older basal part of the roots to prevent the backflow of water (Steudle & Jeschke, 1983; Steudle & Peterson, 1998; Steudle, 2000b). At the same time, the root tip continuously grows into deeper wet soil layers searching for water. It has been described that the maximum radial water uptake in barley roots occurs through this weakly suberized younger zone that includes the root tip, whereas water uptake is significantly decreased in the strongly suberized basal part of the root (Sanderson, 1983; Ranathunge *et al*., 2017). Our measured water and solute permeability values under control conditions with the root pressure probe (Table 1) are perfectly in line with earlier measured values of barely roots in different studies (Knipfer & Fricke, 2010, 2011; Ranathunge *et al*., 2017).

In response to osmotic stress, there was a 2.5‐fold decrease in overall hydrostatic hydraulic conductivity (Lp_r_) of barley roots (Table 1), which correlated well with the significant increase in aliphatic suberin amounts. This stress‐induced aliphatic suberin, which is composed of hydrophobic monomers, markedly reduced the water flow through the apoplast. However, surprisingly, the measured osmotic Lp_r_ through the cell‐to‐cell path, which is mainly facilitated by the plasma membrane‐bound aquaporins (Peterson & Cholewa, 1998; Steudle & Peterson, 1998; Steudle, 2000a,b; Steudle & Ranathunge, 2007; Maurel *et al*., 2015; Gambetta *et al*., 2017), was not curtailed by the rapid development of suberin lamellae and increased suberization of the endodermis under osmotic stress conditions (Table 1). This effect has, until now, only been reported in roots of aeroponic grown maize (Zimmermann *et al*., 2000). Although, in controls, the expression of barley aquaporin genes in roots was much higher than that of suberin biosynthesis genes (Table S4), especially the PIP and TIP aquaporin family members, which are associated with water transport (Maurel *et al*., 2015), the majority of barley aquaporin genes were not differentially regulated in response to osmotic stress. Some of the aquaporin genes were slightly upregulated and other genes were slightly downregulated (Table S4). This supports our results of root osmotic water permeability indicating that the cell‐to‐cell pathway was not affected by osmotic stress. In previous studies, it has been shown that the effect of aquaporins on osmotic stress varies and is highly dependent on the plant species and experimental conditions. The gene expression of some aquaporins was upregulated, but some were downregulated and others were not affected at all (Aroca *et al*., 2012; Gambetta *et al*., 2017). It has been reported previously that post‐transcriptional mechanisms, such as phosphorylation/dephosphorylation and membrane internalization of aquaporins, play a role in the short‐term response (within hours) of barley roots to salinity/osmotic stress (Kaneko *et al*., 2015). By contrast, our data show the adaptation of barley within 6 d of osmotic stress. This suggests that quick short‐term reaction and long‐term adaptation may be different from each other. In the long term, changes in root morphology, including enhanced suberin in the endodermis, have an effect on Lp_r_ in barley roots.

To obtain further insights into an understanding of the drought response, in general highly and successfully drought‐adapted plants are of interest. In roots of *Agave deserti*, which experience prolonged drought of several months or even years, it has been described that the endodermis matures much more rapidly with an accelerated suberization, in which suberin lamellae are deposited close to the root apex (North & Nobel, 1998, 2000). In addition, root growth stops and Lp_r_ is decreased by 62%. Following rewatering of these plants, roots start to elongate again and new lateral roots emerge, which are hardly suberized, and thus these new roots preferentially enhance water uptake. These strategies of a highly drought‐adapted cactus could also be partially applicable for the recovery of drought‐exposed barley seminal roots.

In conclusion, this multifaceted study showed that water deficit, mimicked by different osmotic potentials through PEG 8000 treatment, markedly upregulated the suberin biosynthesis genes in barley seminal roots. By contrast, there was no or minimal effect on the expression of aquaporin genes, which are the regulatory components of water transport through the plasma membrane. The upregulation of suberin biosynthesis genes resulted in an increased endodermal suberization, thus reducing water movements through the apoplastic cell walls to prevent uncontrolled water losses from the root to the dry soil/medium. By contrast, water transport through the cell‐to‐cell path remained unaffected, and thus maintained further efficient water uptake from the soil into the central cylinder of the root. In the future, barley mutants might help to identify further suberin genes and to verify their functions. This could help us to better understand how altered suberin compositions and amounts in roots affect/regulate water and solute transport, and will aid in the improvement of future breeding programs to develop drought‐tolerant barley cultivars.

## Author contributions

T.K. and N.S. performed microscopy. T.K., N.S. and V.V.Z‐D. performed and analyzed the gas chromatography experiments. T.K., A.O., P.Y. and J.A.B. performed and analyzed the RNA‐Seq experiments. T.K. and K.R. performed root pressure probe experiments. L.S., K.R. and F.H. designed and supervised the experiments. T.K., K.R. and L.S. wrote the manuscript. All authors read and approved the final manuscript.

## Supporting information

Please note: Wiley Blackwell are not responsible for the content or functionality of any Supporting Information supplied by the authors. Any queries (other than missing material) should be directed to the *New Phytologist* Central Office.

**Fig. S1** Amounts of aromatic monomers in barley seminal roots grown under control conditions or at a water potential of −0.4, −0.8 or −1.2 MPa.
**Fig. S2** Relative amounts of aliphatic suberin monomers in barley seminal roots grown under control conditions or at a water potential of −0.4, −0.8 or −1.2 MPa.
**Fig. S3** Hypothetical pathway for **s**uberin biosynthesis in barley roots in response to osmotic stress.

**Table S1** Complete list of transcript per million (TPM) values

**Table S2** Complete list of differentially expressed genes

**Table S3** Cross‐comparison of enriched gene ontology (GO) terms amongst differentially expressed genes in the barley seminal root zones A, B and C in response to osmotic stress

**Table S4** Differentially expressed genes (DEGs) and transcript per million (TPM) values of barley suberin, aquaporin, lignin and fatty acid elongation genes

## References

[nph15351-bib-0001] Aroca R , Porcel R , Ruiz‐Lozano JM . 2012 Regulation of root water uptake under abiotic stress conditions. Journal of Experimental Botany 63: 43–57. 21914658 10.1093/jxb/err266

[nph15351-bib-0002] Barberon M , Vermeer JEM , De Bellis D , Wang P , Naseer S , Andersen TG , Humbel BM , Nawrath C , Takano J , Salt DE *et al* 2016 Adaptation of root function by nutrient‐induced plasticity of endodermal differentiation. Cell 164: 447–459. 26777403 10.1016/j.cell.2015.12.021

[nph15351-bib-0003] Benjamini Y , Hochberg Y . 1995 Controlling the false discovery rate: a practical and powerful approach to multiple testing. Journal of the Royal Statistical Society 57: 289–300.

[nph15351-bib-0004] Bernards MA . 2002 Demystifying suberin. Canadian Journal of Botany 80: 227–240.

[nph15351-bib-0005] Boyer JS . 1982 Plant productivity and environment. Science 218: 443–448. 17808529 10.1126/science.218.4571.443

[nph15351-bib-0006] Brundrett MC , Enstone DE , Peterson CA . 1988 A berberine‐aniline blue fluorescent staining procedure for suberin, lignin, and callose in plant tissue. Protoplasma 146: 133–142.

[nph15351-bib-0007] Brundrett MC , Kendrick B , Peterson CA . 1991 Efficient lipid staining in plant material with Sudan red 7B or fluorol yellow 088 in polyethylene glycol‐glycerol. Biotechnic & Histochemistry 66: 111–116. 1716161 10.3109/10520299109110562

[nph15351-bib-0008] Carpita NC . 1996 Structure and biogenesis of the cell walls of grasses. Annual Review of Plant Physiology and Plant Molecular Biology 47: 445–476. 10.1146/annurev.arplant.47.1.445 15012297

[nph15351-bib-0009] Colmer TD , Flowers TJ , Munns R . 2006 Use of wild relatives to improve salt tolerance in wheat. Journal of Experimental Botany 57: 1059–1078. 16513812 10.1093/jxb/erj124

[nph15351-bib-0010] Deng W , Nickle DC , Learn GH , Maust B , Mullins JI . 2007 ViroBLAST: a stand‐alone BLAST web server for flexible queries of multiple databases and user's datasets. Bioinformatics 23: 2334–2336. 17586542 10.1093/bioinformatics/btm331

[nph15351-bib-0011] Enstone DE , Peterson CA , Ma F . 2002 Root endodermis and exodermis: structure, function, and responses to the environment. Journal of Plant Growth Regulation 21: 335–351.

[nph15351-bib-0012] Fraser CM , Chapple C . 2011 The phenylpropanoid pathway in *Arabidopsis* . Arabidopsis Book 9: e0152. 22303276 10.1199/tab.0152 PMC3268504

[nph15351-bib-0013] Frolov A , Bilova T , Paudel G , Berger R , Balcke GU , Birkemeyer C , Wessjohann LA . 2017 Early responses of mature *Arabidopsis thaliana* plants to reduced water potential in the agar‐based polyethylene glycol infusion drought model. Journal of Plant Physiology 208: 70–83. 27889524 10.1016/j.jplph.2016.09.013

[nph15351-bib-0014] Gambetta GA , Knipfer T , Fricke W , McElrone AJ . 2017 Aquaporins and root water uptake. In: Chaumont F, Tyerman SD, eds. Plant aquaporins. Cham, Switzerland: Springer International Publishing, 133–153.

[nph15351-bib-0015] Graça J . 2015 Suberin: the biopolyester at the frontier of plants. Frontiers in Chemistry 3: 62. 26579510 10.3389/fchem.2015.00062 PMC4626755

[nph15351-bib-0016] Hose E , Clarkson DT , Steudle E , Schreiber L , Hartung W . 2001 The exodermis: a variable apoplastic barrier. Journal of Experimental Botany 52: 2245–2264. 11709575 10.1093/jexbot/52.365.2245

[nph15351-bib-0017] Kaneko T , Horie T , Nakahara Y , Tsuji N , Shibasaka M , Katsuhara M . 2015 Dynamic regulation of the root hydraulic conductivity of barley plants in response to salinity/osmotic stress. Plant and Cell Physiology 56: 875–882. 25634964 10.1093/pcp/pcv013

[nph15351-bib-0018] Karahara I , Shibaoka H . 1992 Isolation of Casparian strips from pea roots. Plant and Cell Physiology 33: 555–561.

[nph15351-bib-0019] Kersey PJ , Allen JE , Armean I , Boddu S , Bolt BJ , Carvalho‐Silva D , Christensen M , Davis P , Falin LJ , Grabmueller C *et al* 2016 Ensembl Genomes 2016: more genomes, more complexity. Nucleic Acids Research 44: D574–D580. 26578574 10.1093/nar/gkv1209 PMC4702859

[nph15351-bib-0020] Knipfer T , Fricke W . 2010 Root pressure and a solute reflection coefficient close to unity exclude a purely apoplastic pathway of radial water transport in barley (*Hordeum vulgare*). New Phytologist 187: 159–170. 20412443 10.1111/j.1469-8137.2010.03240.x

[nph15351-bib-0021] Knipfer T , Fricke W . 2011 Water uptake by seminal and adventitious roots in relation to whole‐plant water flow in barley (*Hordeum vulgare* L.). Journal of Experimental Botany 62: 717–733. 20974734 10.1093/jxb/erq312 PMC3003818

[nph15351-bib-0022] Kolattukudy PE , Agrawal VP . 1974 Structure and composition of aliphatic constituents of potato tuber skin (suberin). Lipids 9: 682–691.

[nph15351-bib-0023] Kolattukudy PE , Kronman K , Poulose AJ . 1975 Determination of structure and composition of suberin from the roots of carrot, parsnip, rutabaga, turnip, red beet, and sweet potato by combined gas‐liquid chromatography and mass spectrometry. Plant Physiology 55: 567–573. 16659124 10.1104/pp.55.3.567 PMC541660

[nph15351-bib-0024] Kosová K , Vítámvás P , Prášil IT . 2014 Wheat and barley dehydrins under cold, drought, and salinity – what can LEA‐II proteins tell us about plant stress response? Frontiers in Plant Science 5: 343. 25071816 10.3389/fpls.2014.00343 PMC4089117

[nph15351-bib-0025] Kotula L , Schreiber L , Colmer TD , Nakazono M . 2017 Anatomical and biochemical characterisation of a barrier to radial O_2_ loss in adventitious roots of two contrasting *Hordeum marinum* accessions. Functional Plant Biology 44: 845. 10.1071/FP16327 32480613

[nph15351-bib-0026] Kramer and Boyer . 1995 Water relations of plants and soils. San Diego, CA, USA: Academic Press.

[nph15351-bib-0027] Kreszies T , Schreiber L , Ranathunge K . 2018 Suberized transport barriers in *Arabidopsis*, barley and rice roots: from the model plant to crop species. Journal of Plant Physiology. doi: 10.1016/j.jplph.2018.02.002. 29449027

[nph15351-bib-0028] Krishnamurthy P , Ranathunge K , Franke R , Prakash HS , Schreiber L , Mathew MK . 2009 The role of root apoplastic transport barriers in salt tolerance of rice (*Oryza sativa* L.). Planta 230: 119–134. 19363620 10.1007/s00425-009-0930-6

[nph15351-bib-0029] Krishnamurthy P , Ranathunge K , Nayak S , Schreiber L , Mathew MK . 2011 Root apoplastic barriers block Na^+^ transport to shoots in rice (*Oryza sativa* L.). Journal of Experimental Botany 62: 4215–4228. 21558150 10.1093/jxb/err135 PMC3153681

[nph15351-bib-0030] Lanoue A , Burlat V , Henkes GJ , Koch I , Schurr U , Röse USR . 2010 *De novo* biosynthesis of defense root exudates in response to *Fusarium* attack in barley. New Phytologist 185: 577–588. 19878462 10.1111/j.1469-8137.2009.03066.x

[nph15351-bib-0031] Law CW , Chen Y , Shi W , Smyth GK . 2014 voom: precision weights unlock linear model analysis tools for RNA‐seq read counts. Genome Biology 15: R29. 24485249 10.1186/gb-2014-15-2-r29 PMC4053721

[nph15351-bib-0032] Li‐Beisson Y , Shorrosh B , Beisson F , Andersson MX , Arondel V , Bates PD , Baud S , Bird D , DeBono A , Durrett TP *et al* 2013 Acyl‐lipid metabolism. Arabidopsis Book 11: e0161. 23505340 10.1199/tab.0161 PMC3563272

[nph15351-bib-0033] Lulai ECE , Corsini DL , Crop N . 1998 Differential deposition of suberin phenolic and aliphatic domains and their roles in resistance to infection during potato tuber (*Solanum tuberosum* L.) wound‐healing. Physiological and Molecular Plant Pathology 53: 209–222.

[nph15351-bib-0034] Lupoi JS , Singh S , Parthasarathi R , Simmons BA , Henry RJ . 2015 Recent innovations in analytical methods for the qualitative and quantitative assessment of lignin. Renewable and Sustainable Energy Reviews 49: 871–906.

[nph15351-bib-0035] Man Y , Zhao Y , Ye R , Lin J , Jing Y . 2018 In vivo cytological and chemical analysis of Casparian strips using stimulated Raman scattering microscopy. Journal of Plant Physiology 220: 136–144. 29175545 10.1016/j.jplph.2017.11.002

[nph15351-bib-0036] Mascher M , Gundlach H , Himmelbach A , Beier S , Twardziok SO , Wicker T , Radchuk V , Dockter C , Hedley PE , Russell J *et al* 2017 A chromosome conformation capture ordered sequence of the barley genome. Nature 544: 427–433. 28447635 10.1038/nature22043

[nph15351-bib-0037] Maurel C , Boursiac Y , Luu D‐T , Santoni V , Shahzad Z , Verdoucq L . 2015 Aquaporins in plants. Physiological Reviews 95: 1321–1358. 26336033 10.1152/physrev.00008.2015

[nph15351-bib-0038] Melillo JM , Richmond T , Yohe GW . 2014 Highlights of climate change impacts in the United States: The Third National Climate Assessment. Washington, DC, USA: US Government Printing Office, US Global Change Research Program.

[nph15351-bib-0039] Michel BE . 1983 Evaluation of the water potentials of solutions of Polyethylene Glycol 8000 both in the absence and presence of other solutes. Plant Physiology 72: 66–70. 16662983 10.1104/pp.72.1.66 PMC1066170

[nph15351-bib-0040] Naseer S , Lee Y , Lapierre C , Franke R , Nawrath C , Geldner N . 2012 Casparian strip diffusion barrier in Arabidopsis is made of a lignin polymer without suberin. Proceedings of the National Academy of Sciences, USA 109: 10101–10106. 10.1073/pnas.1205726109 PMC3382560 22665765

[nph15351-bib-0041] North GB , Nobel PS . 1998 Water uptake and structural plasticity along roots of a desert succulent during prolonged drought. Plant, Cell & Environment 21: 705–713.

[nph15351-bib-0042] North GB , Nobel PS . 2000 Heterogeneity in water availability alters cellular development and hydraulic conductivity along roots of a desert succulent. Annals of Botany 85: 247–255.

[nph15351-bib-0043] Opitz N , Marcon C , Paschold A , Malik WA , Lithio A , Brandt R , Piepho H‐P , Nettleton D , Hochholdinger F . 2016 Extensive tissue‐specific transcriptomic plasticity in maize primary roots upon water deficit. Journal of Experimental Botany 67: 1095–1107. 26463995 10.1093/jxb/erv453 PMC4753846

[nph15351-bib-0044] Peterson CA , Cholewa E . 1998 Structural modifications of the apoplast and their potential impact on ion uptake. Zeitschrift für Pflanzenernährung und Bodenkunde 161: 521–531.

[nph15351-bib-0045] Ranathunge K , Kim YX , Wassmann F , Kreszies T , Zeisler V , Schreiber L . 2017 The composite water and solute transport of barley (*Hordeum vulgare*) roots: effect of suberized barriers. Annals of Botany 119: 629–643. 28065927 10.1093/aob/mcw252 PMC5604597

[nph15351-bib-0046] Ranathunge K , Lin J , Steudle E , Schreiber L . 2011a Stagnant deoxygenated growth enhances root suberization and lignifications, but differentially affects water and NaCl permeabilities in rice (*Oryza sativa* L.) roots. Plant, Cell & Environment 34: 1223–1240. 10.1111/j.1365-3040.2011.02318.x 21414017

[nph15351-bib-0047] Ranathunge K , Schreiber L , Franke R . 2011b Suberin research in the genomics era – new interest for an old polymer. Plant Science 180: 399–413. 21421386 10.1016/j.plantsci.2010.11.003

[nph15351-bib-0048] Ranathunge K , Schreiber L . 2011 Water and solute permeabilities of Arabidopsis roots in relation to the amount and composition of aliphatic suberin. Journal of Experimental Botany 62: 1961–1974. 21421706 10.1093/jxb/erq389 PMC3060681

[nph15351-bib-0049] Ranathunge K , Schreiber L , Bi Y‐M , Rothstein SJ . 2016 Ammonium‐induced architectural and anatomical changes with altered suberin and lignin levels significantly change water and solute permeabilities of rice (*Oryza sativa* L.) roots. Planta 243: 231–249. 26384983 10.1007/s00425-015-2406-1

[nph15351-bib-0050] Ranathunge K , Thomas RH , Fang X , Peterson CA , Gijzen M , Bernards MA . 2008 Soybean root suberin and partial resistance to root rot caused by *Phytophthora sojae* . Phytopathology 98: 1179–1189. 18943406 10.1094/PHYTO-98-11-1179

[nph15351-bib-0051] Sanderson J . 1983 Water uptake by different regions of the barley root. Pathways of radial flow in relation to development of the endodermis. Journal of Experimental Botany 34: 240–253.

[nph15351-bib-0052] Schreiber L . 1996 Chemical composition of Casparian strips isolated from *Clivia miniata* Reg. roots: evidence for lignin. Planta 199: 596–601.

[nph15351-bib-0053] Schreiber L , Breiner H‐W , Riederer M , Düggelin M , Guggenheim R . 1994 The Casparian strip of *Clivia miniata* Reg. roots: isolation, fine structure and chemical nature. Botanica Acta 107: 353–361.

[nph15351-bib-0054] Schreiber L , Franke R , Hartmann KD , Ranathunge K , Steudle E . 2005 The chemical composition of suberin in apoplastic barriers affects radial hydraulic conductivity differently in the roots of rice (*Oryza sativa* L. cv. IR64) and corn (*Zea mays* L. cv. Helix). Journal of Experimental Botany 56: 1427–1436. 15809280 10.1093/jxb/eri144

[nph15351-bib-0055] Schreiber L , Hartmann K , Skrabs M , Zeier J . 1999 Apoplastic barriers in roots: chemical composition of endodermal and hypodermal cell walls. Journal of Experimental Botany 50: 1267–1280.

[nph15351-bib-0056] Shiono K , Ando M , Nishiuchi S , Takahashi H , Watanabe K , Nakamura M , Matsuo Y , Yasuno N , Yamanouchi U , Fujimoto M *et al* 2014a RCN1/OsABCG5, an ATP‐binding cassette (ABC) transporter, is required for hypodermal suberization of roots in rice (*Oryza sativa*). Plant Journal 80: 40–51. 25041515 10.1111/tpj.12614

[nph15351-bib-0057] Shiono K , Yamauchi T , Yamazaki S , Mohanty B , Malik AI , Nagamura Y , Nishizawa NK , Tsutsumi N , Colmer TD , Nakazono M . 2014b Microarray analysis of laser‐microdissected tissues indicates the biosynthesis of suberin in the outer part of roots during formation of a barrier to radial oxygen loss in rice (*Oryza sativa*). Journal of Experimental Botany 65: 4795–4806. 24913626 10.1093/jxb/eru235

[nph15351-bib-0058] Smyth GK . 2004 Linear models and empirical Bayes methods for assessing differential expression in microarray experiments. Statistical Applications in Genetics and Molecular Biology 3: 1–25. 10.2202/1544-6115.1027 16646809

[nph15351-bib-0059] Smyth GK . 2005 limma: linear models for microarray data In: Gentlemen R, Carey V, Dudoit S, Irizarry R, Huber W, eds. Bioinformatics and computational biology solutions using R and Bioconductor. New York, NY, USA: Springer‐Verlag, 397–420.

[nph15351-bib-0060] Steudle E . 2000a Water uptake by roots: an integration of views. Plant and Soil 226: 45–56.

[nph15351-bib-0061] Steudle E . 2000b Water uptake by roots: effects of water deficit. Journal of Experimental Botany 51: 1531–1542. 11006304 10.1093/jexbot/51.350.1531

[nph15351-bib-0062] Steudle E , Jeschke WD . 1983 Water transport in barley roots. Planta 158: 237–248. 24264613 10.1007/BF01075260

[nph15351-bib-0063] Steudle E , Oren R , Schulze E‐D . 1987 Water transport in maize roots: measurement of hydraulic conductivity, solute permeability, and of reflection coefficients of excised roots using the root pressure probe. Plant Physiology 84: 1220–1232. 16665588 10.1104/pp.84.4.1220 PMC1056755

[nph15351-bib-0064] Steudle E , Peterson CA . 1998 How does water get through roots? Journal of Experimental Botany 49: 775–788.

[nph15351-bib-0065] Steudle E , Ranathunge K . 2007 Apoplastic water transport in roots In: Sattelmacher B, Horst WJ, eds. The apoplast of higher plants: compartment of storage, transport and reactions. Dordrecht, the Netherlands: Springer, 119–130.

[nph15351-bib-0066] Thomas R , Fang X , Ranathunge K , Anderson TR , Peterson CA , Bernards MA . 2007 Soybean root suberin: anatomical distribution, chemical composition, and relationship to partial resistance to *Phytophthora sojae* . Plant Physiology 144: 299–311. 17494920 10.1104/pp.106.091090 PMC1913776

[nph15351-bib-0067] Tian T , Liu Y , Yan H , You Q , Yi X , Du Z , Xu W , Su Z . 2017 agriGO v2.0: a GO analysis toolkit for the agricultural community, 2017 update. Nucleic Acids Research 45: W122–W129. 28472432 10.1093/nar/gkx382 PMC5793732

[nph15351-bib-0068] Tylová E , Pecková E , Blascheová Z , Soukup A . 2017 Casparian bands and suberin lamellae in exodermis of lateral roots: an important trait of roots system response to abiotic stress factors. Annals of Botany 120: 71–85. 28605408 10.1093/aob/mcx047 PMC5737840

[nph15351-bib-0069] Verslues PE , Agarwal M , Katiyar‐Agarwal S , Zhu J , Zhu J‐K . 2006 Methods and concepts in quantifying resistance to drought, salt and freezing, abiotic stresses that affect plant water status. Plant Journal 45: 523–539. 16441347 10.1111/j.1365-313X.2005.02593.x

[nph15351-bib-0070] Vishwanath SJ , Delude C , Domergue F , Rowland O . 2015 Suberin: biosynthesis, regulation, and polymer assembly of a protective extracellular barrier. Plant Cell Reports 34: 573–586. 25504271 10.1007/s00299-014-1727-z

[nph15351-bib-0071] Wagner GP , Kin K , Lynch VJ . 2012 Measurement of mRNA abundance using RNA‐seq data: RPKM measure is inconsistent among samples. Theory in Biosciences 131: 281–285. 22872506 10.1007/s12064-012-0162-3

[nph15351-bib-0072] Yamaguchi M , Sharp RE . 2010 Complexity and coordination of root growth at low water potentials: recent advances from transcriptomic and proteomic analyses. Plant, Cell & Environment 33: 590–603. 10.1111/j.1365-3040.2009.02064.x 19895398

[nph15351-bib-0073] Zeier J , Ruel K , Ryser U , Schreiber L . 1999 Chemical analysis and immunolocalisation of lignin and suberin in endodermal and hypodermal/rhizodermal cell walls of developing maize (*Zea mays* L.) primary roots. Planta 209: 1–12. 10467026 10.1007/s004250050601

[nph15351-bib-0074] Zeier J , Schreiber L . 1997 Chemical composition of hypodermal and endodermal cell walls and xylem vessels isolated from *Clivia miniata* . Plant Physiology 113: 1223–1231. 12223670 10.1104/pp.113.4.1223 PMC158245

[nph15351-bib-0075] Zeier J , Schreiber L . 1998 Comparative investigation of primary and tertiary endodermal cell walls isolated from the roots of five monocotyledonous species: chemical composition in relation to fine structure. Planta 206: 349–361.

[nph15351-bib-0076] Zimmermann HM , Hartmann K , Schreiber L , Steudle E . 2000 Chemical composition of apoplastic transport barriers in relation to radial hydraulic conductivity of corn roots (*Zea mays* L.). Planta 210: 302–311. 10664137 10.1007/PL00008138

[nph15351-bib-0077] Zingaretti SM , Inácio MC , de Matos Pereira L , Antunes Paz T , de Castro França S . 2013 Water stress and agriculture In: Akinci DS, ed. Responses of organisms to water stress. London, UK: InTechOpen, 151–179.

