# Supplementary material for: Osmotic stress enhances suberization of apoplastic barriers in barley seminal roots: analysis of chemical, transcriptomic and physiological responses
Source: New Phytol. 2018 Jul 28;221(1):180–94. doi: 10.1111/nph.15351 (PMC6586163; doi:10.1111/nph.15351)
Supplement: Supplementary file 1 — Fig. S1 Amounts of aromatic monomers in barley seminal roots grown under control conditions or at a water potential of −0.4, −0.8 or −1.2 MPa. Fig. S2 Relative amounts of aliphatic suberin monomers in barley seminal roots grown under control conditions or at a water potential of −0.4, −0.8 or −1.2 MPa. Fig. S3 Hypothetical pathway for suberin biosynthesis in barley roots in response to osmotic stress. [file NPH-221-180-s001.pdf]

***New Phytologist* Supporting Information**

Article title: Osmotic stress enhances suberization of apoplastic barriers in barley seminal roots: analysis of chemical, transcriptomic and physiological responses

Authors: Tino Kreszies[1*], Nandhini Shellakkutti[1], Alina Osthoff[2], Peng Yu[2], Jutta A. Baldauf[2], Viktoria V. Zeisler-Diehl[1], Kosala Ranathunge[3], Frank Hochholdinger[2], and Lukas Schreiber[1]
Article acceptance date: 18 June 2018

The following Supporting Information is available for this article:

Fig. S1: Amounts of aromatic monomers in barley seminal roots grown under control conditions or at water potentials of -0.4 MPa, -0.8 MPa or -1.2 MPa.

Fig. S2: Relative amount of aliphatic suberin monomers in barley seminal roots grown under control conditions or at water potentials of -0.4 MPa, -0.8 MPa or -1.2 MPa.

Fig. S3: Hypothetical pathway for **s**uberin biosynthesis in barley roots in response to osmotic stress.

Table S1: Complete list of transcript per million values (TPM).

Table S2: Complete list of differentially expressed genes.

Table S3: Cross comparison of enriched GO terms among differentially expressed genes in the barley seminal root zones A, B and C in response to osmotic stress.

Table S4: DEG and TPM values of barley suberin, aquaporin, lignin and fatty acid elongation genes.

Fig. S1 Amounts of aromatic monomers in barley seminal roots grown under control conditions or at water potential of -0.4 MPa, -0.8 MPa or -1.2 MPa.
For chemical analysis the roots were divided into three root zones from the apical root tip Zone A over Zone B to the basal part Zone C (Fig. 1). The bars represent mean values with standard deviation of three biological replicates.

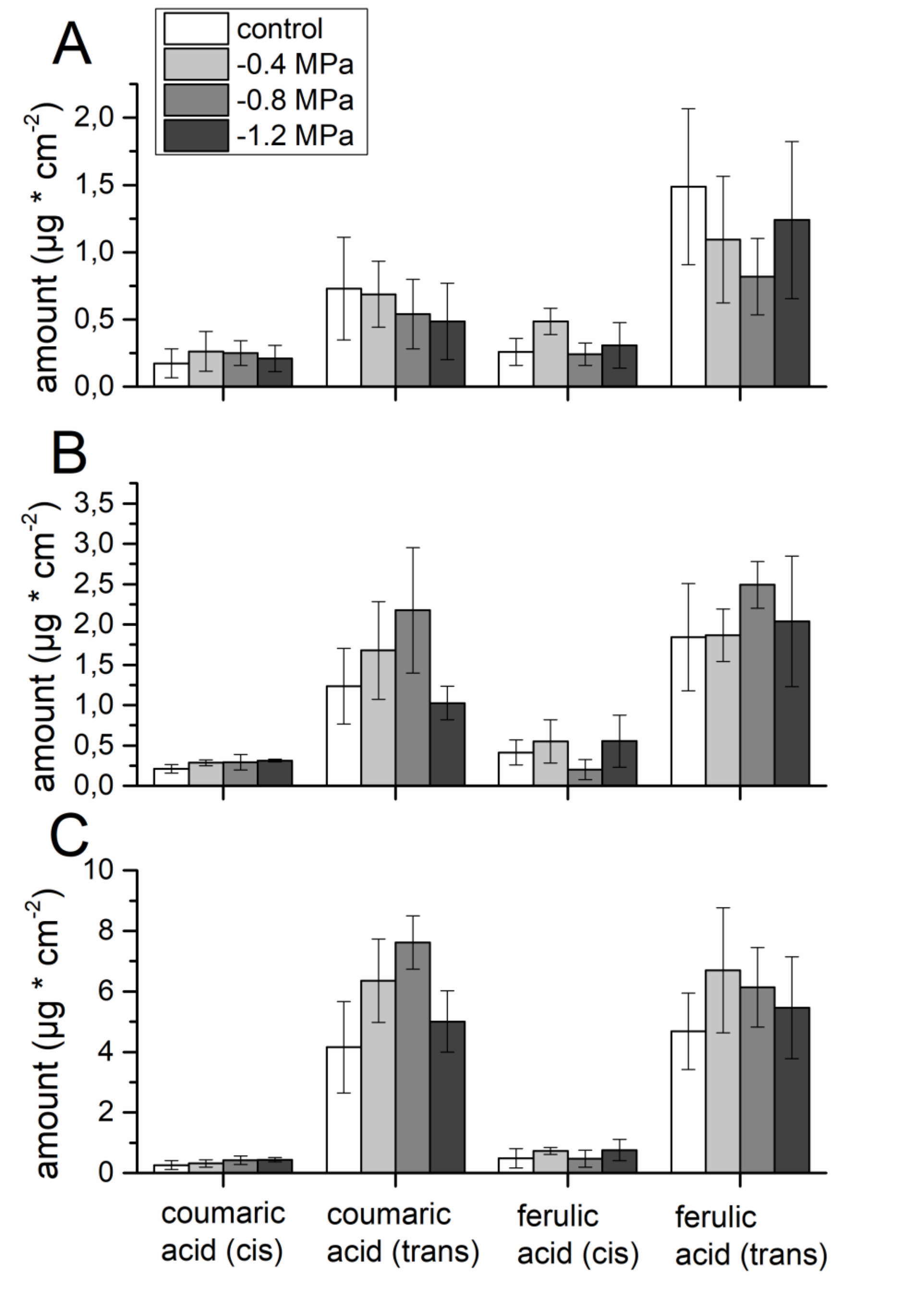

Fig. S2 Relative amount of aliphatic suberin monomers in barley seminal roots divided in 3 root zones (Fig. 1) grown under control conditions or at water potentials of -0.4 MPa, -0.8 MPa and -1.2 MPa. Alc = alcohol; FA = fatty acids; diacids = α–ω dicarboxylic acids; w-OH = ω-hydroxyl acids

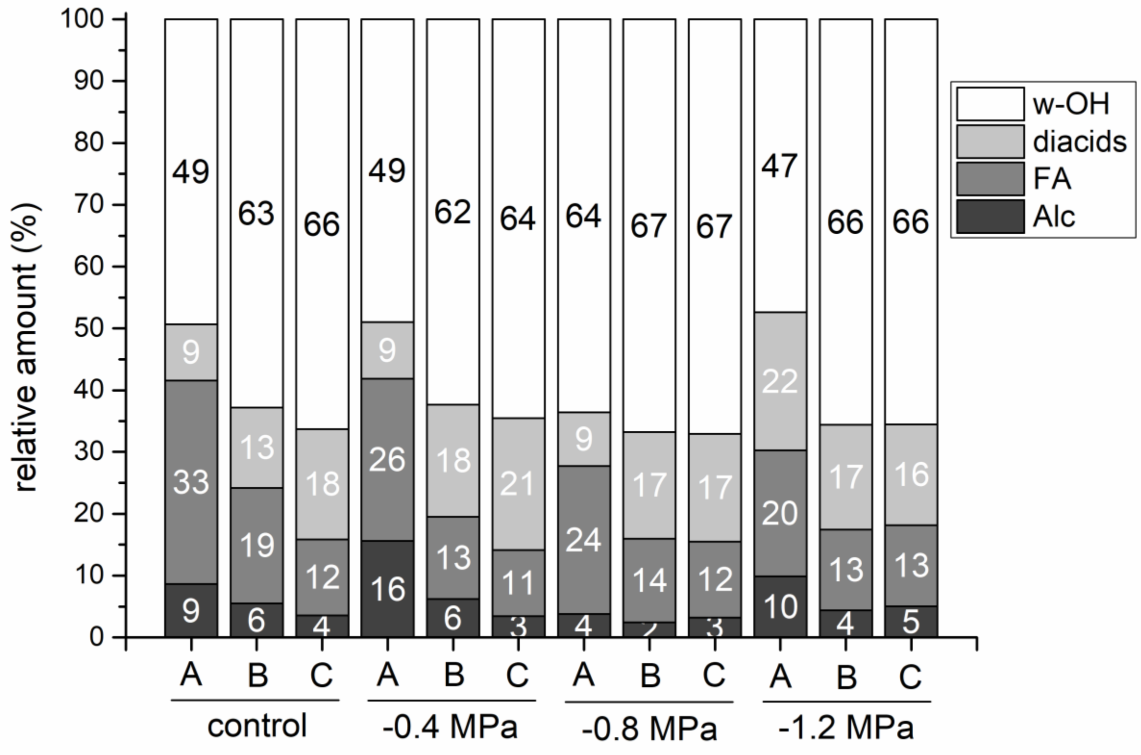

Fig. S3: Hypothetical pathway for **s**uberin biosynthesis in barley roots in response to osmotic stress. Genes in red are up-regulated in barley seminal roots in response to drought stress (Fig. 9).
Cytochromes P450 converting fatty acids into ω-hydroxy acids and α-ω dicarboxylic acids. LACS: Long-Chain Acyl-CoA Synthetases. AlcFAR: Alcohol-forming Fatty Acyl-CoA Reductase. KCS: Ketoacyl-CoA Synthase from the fatty acid elongation complex. Cytochromes P450 synthesize coumaric and ferulic acids. ASFT/BAHD: Aliphatic Suberin Feruloyl Transferase link aliphatic and aromatic suberin monomers to suberin building units.

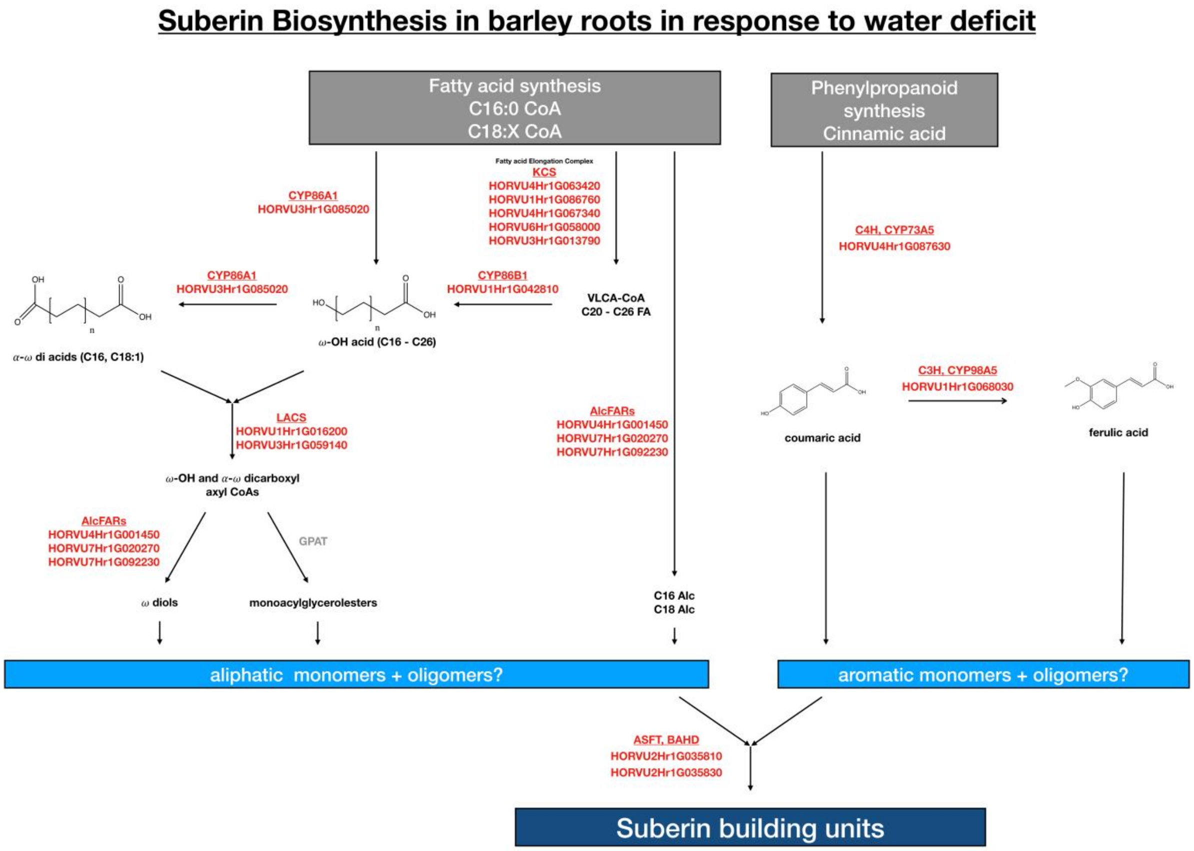

Table S1: Complete list of transcript per million values (TPM).
The Table includes the mean values and standard deviation for the three root zones under control and stress treatment of all barley genes IDs

Table S2: Complete list of differentially expressed genes.
The Table consists of several sheets which include the barley gene IDs with their log2FC and t, P, adjusted P and B value.
Sheets: A_SvsK; B_SvsK; C_SvsK represent differentially expressed genes in the three root Zones A, B and C each stress versus control
Sheets: K_AvsB; K_BvsC; K_AvsC represent differentially expressed genes in control conditions over the length of the root
Sheets: S_AvsB; S_BvsC; S_AvsC represent differentially expressed genes in control conditions over the length of the root

Table S3: Cross comparison of enriched GO terms among differentially expressed genes in the barley seminal root zones A, B and C in response to osmotic stress.
The Table represents the results of the cross comparison of SEA (SEACOMPARE) tool by AgriGOv2.0 as described in material and methods. The sheet "All GO DEG" shows the results of all differently enriched GO while the sheet "GO up and down" separates further between up- and down-regulated enriched GO terms.
The Colour model (CM) shows how small the term's adjusted p-value is. The more significant statistically, the colour is darker and redder. Grey is not significant.

Table S4: DEG and TPM values of barley suberin, aquaporin, lignin and fatty acid elongation genes.
This Table includes DEG and TPM values of putative barley homologues to their respective Arabidopsis gene of suberin, aquaporin, lignin and fatty acid elongation genes.
